# Geriatric Importance of Histopathological Parameters Evaluated in Thyroidectomy Specimens: A Single Center Retrospective Analysis

**DOI:** 10.3390/jpm14010095

**Published:** 2024-01-15

**Authors:** Nesibe Kahraman Çetin, Sinan Can Taşan

**Affiliations:** Department of Pathology, Faculty of Medicine, Aydın Adnan Menderes University, 09010 Aydın, Turkey; sctasan@adu.edu.tr

**Keywords:** thyroidectomy, malignancy, geriatrics, histopathology, age, sex

## Abstract

Nowadays, the aging human population exerts a notable influence on the treatment of thyroid diseases. The most appropriate approach for the treatment of benign and malignant thyroid diseases in older adults has not yet been determined. The aim of our study is to evaluate the effect of thyroidectomies in geriatric patients considering age, sex and histopathological parameters and to determine the importance of thyroidectomy as a treatment option in the geriatric population. A total of 910 cases from all age groups were included, for which thyroidectomies were examined and reported. In accordance with the College of American Pathologists Cancer Protocol for thyroid reporting, considering geriatric patients, the rate of Thyroid Follicular Nodular Disease was significantly higher among the tumor types in the benign tumor group (*p* = 0.033), while Anaplastic Thyroid Carcinoma rate was higher in the malignant tumor group. The diagnosis rate of malignant tumors was higher in males, reflecting a more advanced pT stage (*p* < 0.001), larger tumor size (*p* < 0.001) and increased lymph node involvement rate (*p* = 0.039). Given that increasing age is associated with a heightened incidence of thyroid disease, the safety of surgery for geriatric patients is an important issue. Thyroidectomy should be considered in the treatment of these patients, especially in males, as the rate of malignant diagnosis and worse histopathological parameters are seen with increasing age.

## 1. Introduction

The aging of the human population is a global phenomenon, with the elderly population steadily increasing in number over time. The percentage of geriatric individuals (65 years and older) is expected to increase from 15% to 21% within a decade. In the United States, the geriatric demographic consists of approximately 50 million individuals, and it is anticipated to surge by around 50% by 2030 [1,2]. This trend is echoed in Turkey, with projections suggesting an increase to 10.2% by 2023 and 20.8% by 2050 [3]. As the population ages, both worldwide and in Turkey, and as the prevention and treatment of diseases continue to develop, physicians will face an aging patient population for whom they must suggest the best treatment decisions [4,5]. Considering the increase in the number of geriatric patients, it is necessary to consider and anticipate the difficulties that may arise. Therefore, treatment policies and surgical treatment options should be revised according to patient age, prioritizing common diseases [5].

The aging human population will also affect the treatment of thyroid diseases, which are quite common; therefore, treatment decisions in elderly patients deviate from those made for younger patients [4,5]. Considering age-based differences, the most appropriate approach for the treatment of benign and malignant thyroid diseases in older adults has not yet been determined. Elderly patients are more likely to present with cardiovascular, neurological and metabolic comorbidities, which may increase the intrinsic risks of thyroid surgery and general anesthesia [5,6]. Therefore, clinicians must be familiar with the management of thyroid diseases in geriatric patients in order to optimize the evaluation and management of thyroid nodules [5]. Personalizing risk assessment is particularly important given the wide range of health profiles in elderly patients, especially when considering surgical treatment. Accurate risk assessments and strategies to mitigate outcomes are being explored to optimize surgical outcomes in elderly patients [5,6].

Even now, there is no consensus on whether thyroidectomy is safe in geriatric populations [4]. While some studies have reported higher complication rates in the elderly who underwent thyroidectomy [7,8], others have concluded that there is no increase in the risk of complications [9,10]. In studies examining the effect of age in patients who underwent thyroidectomy, the cohorts were either small or the study scope was narrow, and surgery was less preferred in elderly patients because of the high risk of morbidity [4,7,8,9,10]. In contrast, recent studies have shown a significant reduction in cancer-related morbidity and increased overall survival in elderly patients treated with surgery [11,12]. In addition, the increase in complications in elderly patients is related to biological age and comorbidities rather than chronological age [5,9]. The development of diagnostic techniques, ease of access to hospital facilities, and use of new surgical and anesthesiology techniques reduce the surgical time and incidence of some complications, making thyroidectomy more convenient for geriatric patients [6,7]. Therefore, indications for thyroid surgery are constantly increasing worldwide [7]. Elderly patients with thyroid disorders need to be carefully evaluated, both in terms of their co-morbidities and fitness for surgery and also in terms of the risk derived from the thyroid disease. For the appropriate approach to thyroidectomy in elderly patients with thyroid disease, surgical decision-making should also consider the features of the thyroid disease being treated (5). Before reaching a decision as to whether thyroidectomy is appropriate, the thyroid should be investigated in terms of the histopathological type of the disease and the parameters reported in pathological evaluations that describe the lesion. To date, many studies have evaluated the global incidence and mortality of thyroid cancer; however, the number of studies examining age, sex and histopathological results in different countries is limited [13,14,15]. This study aimed to evaluate thyroidectomies performed in geriatric patients, assessing age, sex and histopathological parameters. By juxtaposing these results with the existing literature, we aimed to determine the pivotal role of thyroidectomy as a treatment option in the geriatric population. This study focused on the results of histopathological parameters evaluated in elderly patients, mainly from thyroidectomies. Understanding the importance of these parameters will be very useful in surgery decision-making as well as the management of the follow-up and treatment of patients.

## 2. Methods

This study was approved by the Aydın Adnan Menderes University Faculty of Medicine Non-Invasive Clinical Research Ethics Committee with protocol number 2023/130.

In this study, we retrospectively examined the pathology archive records of the thyroidectomy materials examined between 1 January 2017 and 1 May 2023 within the Medical Pathology Laboratory of Aydın Adnan Menderes University Faculty of Medicine. We retrieved case data by reviewing the archived pathology reports. Our study included 910 patients from all age groups whose thyroidectomies were investigated and reported. The number and proportions of the cases were determined. They were grouped according to age, sex, surgery procedure, histopathological groups, histopathological types, tumor size, tumor localization, multifocality, lymphovascular invasion, perineural invasion, margin status, extrathyroidal extension, lymph node involvement and pT category (pTNM classification, American Joint Committee on Cancer (AJCC) 8. Edition) [16]. The thyroid histopathological groupings and neoplasia types used in this study were re-evaluated according to the 5th Edition of the World Health Organization (WHO) Classification of Thyroid Tumors [17].

## 3. Statistical Analysis

All statistical analyses were performed with SPSS version 26.0 software (SPSS, Chicago, IL, USA). The Kolmogorov–Smirnov test was used to determine whether the distribution of discrete numerical variables was close to normal. Descriptive statistics, discrete numerical variables, were expressed as mean ± standard deviation, while categorical variables were expressed as the number of cases and (%). The significance of the differences among groups in terms of mean age and tumor size was analyzed by Mann–Whitney U and Kruskal–Wallis Analysis of Variance tests. The 2 × 2 Chi-Square and Pearson Multi-Eye Chi-Square Test were used to analyze the count data. However, if the expected frequency was below 5 in at least ¼ of the cells in the 2 × 2 crosstabs, the categorical data were evaluated with Fisher’s exact probability test; in cases where the expected frequency was between 5 and 25, the continuity-corrected χ^2^ test was used. A multivariate analysis of variance was performed with the MANOVA test. Since the variances were not equal, Tamhane’s T2 Post Hoc Test was used with Wilks’ lambda. Unless otherwise stated, results for *p* < 0.05 were considered statistically significant. However, Bonferroni Correction was performed to control for Type I error in all possible multiple comparisons.

## 4. Results

The ages of the patients diagnosed in the Medical Pathology Department of our hospital over a period of approximately 5.5 years ranged from 11 to 87 years. Age values were not normally distributed (Kolmogorov–Smirnov, *p* < 0.001). The overall mean age was 49.7 (SD.14.4) and the median value was 51. When analyzed according to sex, the average age of females was significantly lower than that of males (*p* < 0.001). The median ages were 54 for males and 50 for females. The age range with the highest concentration was 55–64 for males and both 45–54 and 55–64 for females. A statistically significant difference was observed between the sexes in terms of age range distribution (*p* = 0.004). Within the geriatric group, males constituted 21.4% and females 14.1%; this difference was statistically significant (*p* = 0.014). Tumor size did not exhibit a normal distribution (Kolmogorov–Smirnov, *p* < 0.001). In the malignant neoplastic tumor group, male sex was significantly associated with a more advanced pT stage and larger tumor size (*p* < 0.001). In the group diagnosed with malignant neoplastic tumors, the rate of lymph node involvement was significantly higher in males (25%) than in females (13.8%) (*p* = 0.039). Demographic and histopathological data of the patients are given in Table 1.

When the cases were divided into two subgroups, the geriatric group (aged 65 years and older) and the non-geriatric group (aged under 65 years), the proportion of male patients in the geriatric group and female patients in the non-geriatric group was significantly higher (*p* = 0.010). The mean tumor size in the geriatric group was statistically significantly larger than the mean of the non-geriatric group (*p* < 0.001). Additionally, as a result of a multivariate analysis of variance performed for malignant features between these groups, an advanced pT stage was found in the geriatric group. (*p* = 0.007) There was no statistically significant difference between parameters such as lymphovascular or perineural invasion. The geriatric group accounted for 75% of the most advanced pT4 tumors, and the geriatric group was statistically associated with significantly more advanced pT staging (*p* = 0.001, Table 2).

**Figure 1:**
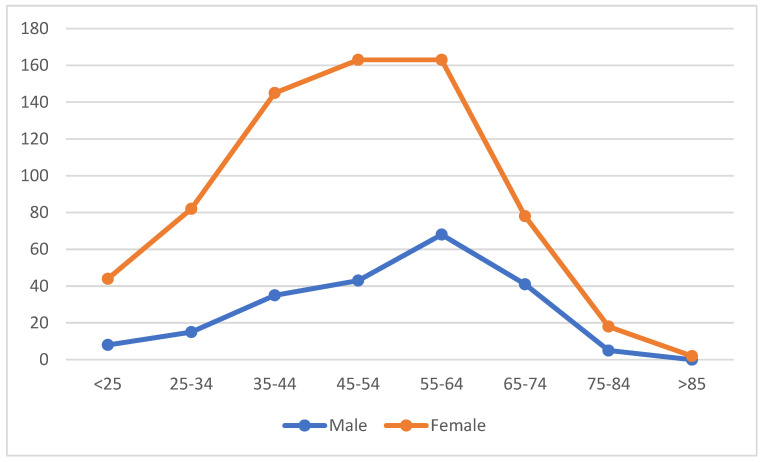
The distribution of age groups by sex.

Patients were divided into eight different age groups according to their density, and the geriatric group was subdivided into three subgroups: young-elderly (65–74 years old), mid-elderly (75–84 years old) and advanced-elderly (85 years and older). The distribution ratio of males was significantly higher in the 65–74 age groups, and the ratio of females was significantly higher in the 25–34 age groups (*p* = 0.004) (Figure 1). No statistically significant difference was observed among the classification of geriatric age groups and genders (*p* = 0.303). However, there were statistically significant differences in the proportional distributions among the age groups regarding extrathyroidal extension, lymph node involvement, margin status and pT staging. Extrathyroidal extension was more common in the 45–54 and 75–84 age ranges than in the other age groups (*p* < 0.001); lymph node involvement was more common in patients under the age of 25 and the 75–84 age range (*p* = 0.005). The incidence of pT3 stage tumors in the age group below 25 years and between the ages of 75 and 84. In the 75–84 age group, the incidence of pT4 stage tumors was higher than in the other groups (*p* < 0.001, Table 3). Margin status was more common in the 75–84 age range than in other age groups (*p* = 0.029).

**Figure 2:**
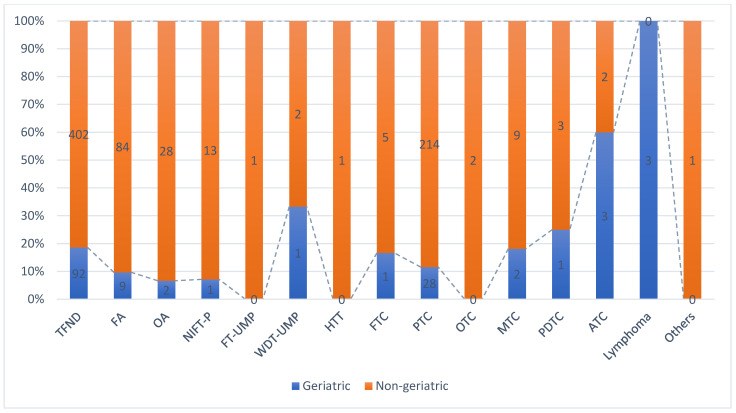
One hundred percent cumulative distribution of histopathological types by geriatric grouping. (TFND: Thyroid Follicular Nodular Disease, FA: Follicular Adenoma, OA: Oncocytic Adenoma, NIFT-P: Noninvasive follicular thyroid neoplasm with papillary-like nuclear features, FT-UMP: Follicular Tumor-Uncertain Malignant Potential WDT-UMP: Well Differentiated Tumor -Uncertain Malignant Potential HTT: Hyalinizing trabecular tumor, FTC: Follicular Thyroid Carcinoma, PTC: Papillary Thyroid Carcinoma, OTC: Oncocytic Thyroid Carcinoma, MTC: Medullary Thyroid Carcinoma, PDTC: Poorly Differentiated Thyroid Carcinoma, ATC: Anaplastic Thyroid Carcinoma).

Histopathological evaluation of the thyroidectomy materials was performed under three main categories, which were divided into various histopathological types [17]. The most common histopathological type among our cases was Thyroid Follicular Nodular Disease (TFND) (494 patients, 54.3%), followed by Papillary Thyroid Carcinoma (PTC) (242 patients, 26.6%) and Follicular Adenoma (FA) (93 patients, 10.2%). Among the cases in the benign tumors group, the proportion of those diagnosed with TFND in the geriatric group was significantly higher than in the non-geriatric group. Further, the proportions of patients diagnosed with FA and Oncocytic Adenoma (OA), which are included in the benign tumors group, was higher in the non-geriatric group (*p* = 0.033). Among cases with a malignant histopathological diagnosis, the rate of PTC in the non-geriatric group and Anaplastic Thyroid Carcinoma (ATC) in the geriatric group was higher. All cases diagnosed with lymphoma belonged to the geriatric group (*p* = 0.001). (Figure 2) The total thyroidectomy rate was higher in patients with a malignant histopathological diagnosis (*p* < 0.001). In patients diagnosed with ATC, which we see more frequently in geriatrics, lymph node involvement (*p* = 0.016), extrathyroidal extension (*p* = 0.001), and perineural invasion (*p* < 0.001) were more common than in the other histopathological types. Among the cases with a staged malignant histopathological diagnosis, 210 were at pT1, 37 at pT2, 21 at pT3 and 4 at pT4. Patients with ATC generally had a more advanced pT stage and larger tumor size than those in the other groups (*p* < 0.001). No significant difference was found between the histopathological types in the low-risk neoplasms and geriatric groups (*p* = 0.243). No significant difference was found between the histopathological types and sex (*p* = 0.055) and multifocality (*p* = 0.056).

## 5. Discussion

The most common causes of thyroid diseases, which demonstrate a higher frequency in conjunction with increasing age, are often associated with suspected thyroid malignancy, resistance to treatment or cosmetic concerns. Treatment modalities for these conditions can include lobectomy, subtotal/total thyroidectomy, postoperative radioiodine therapy, thyroid-stimulating hormone suppressive therapy, as well as new molecular therapies [18]. Thyroid diseases are generally seen more frequently in females, with a female/male ratio of approximately 3.3/1 [13]. In our study, most patients who underwent thyroidectomy were female, and the female/male ratio was approximately 3.2. The mean age of thyroidectomy was significantly lower in females. Higher incidence rates were observed in the 4th–5th decades of life for females and in the 5th–6th decades of life for males (*p* = 0.004). This may be attributed to the fact that thyroid diseases are more common in females, constituting a potential etiological factor. Moreover, surgical intervention is often favored and implemented more readily due to the earlier onset of disease manifestation in females.

Various classifications, staging systems and reporting protocols have been used for histopathological examination of thyroidectomies. The purpose of this is primarily to facilitate communication between doctors and institutions by using common identifiers and to provide standardization. This approach also enhances the creation of treatment and follow-up plans and prognostic estimations for patients. Further, it provides the necessary dataset for retrospective clinical studies [19]. Currently, the most frequently used staging system for thyroidectomies is the pTNM classification AJCC 8. Edition and the College of American Pathologists Cancer Protocol (CAPCP) is frequently used to ensure that all pathology reports contain the data items necessary to improve patient care [16,20]. TNM classification is currently widely used in research or epidemiological studies and in determining the risk of mortality and is recommended by the European Thyroid Association (ETA) and the American Thyroid Association (ATA) [20]. The CAPCP Thyroid Reporting Protocol is the most comprehensive and regularly updated protocol for reviewing material from patients with thyroid carcinomas. Thus, it provides clinicians with the necessary information for treatment decisions such as cancer staging, risk stratification, completion of thyroidectomy, and the need for radioactive iodine ablation. Mandatory features to be reported in the pathology report according to the CAPCP thyroid reporting protocol: surgical procedure; tumor focality; tumor localization; tumor size; histopathological type, margin status, angioinvasion; lymphatic invasion; perineural invasion; extrathyroidal extension; number of lymph nodes examined and involved, if any; largest metastatic lymph node size; extra nodal spread; and pTNM classification (AJCC/TNM 8th Edition) [16].

As people age, the frequency of benign and malignant thyroid diseases increases [4,21]. In approximately 50% of geriatric patients, thyroid nodules are detected on an ultrasound [21]. TFND is present in approximately 90% of females over the age of 70 and 60% of males over the age of 80 (8). Thyroid cancer is the most common endocrine malignancy, with PTC being the most frequent, mainly in the 5th decade of life [12,13]. Although the incidence of thyroid nodules is higher in elderly patients in the literature, the incidence of thyroid cancer is generally lower [1,5]. However, many studies have reported that if thyroid cancer is diagnosed, it tends to be a high-risk histopathological type with a more aggressive clinical course [1,21]. Some subtypes of malignant thyroid diseases, such as ATC and primary thyroid lymphoma, occur in patients over the age of 60, especially [5,21]. Differentiated carcinomas are generally more aggressive in pTNM after 55 years of age [22]. Therefore, thyroid nodules require careful examination in elderly patients in terms of their histopathological types and parameters [21]. Considering the distribution of histopathological diagnoses in the geriatric age group in our study, the rate of TNFD was higher in the benign tumors group (*p* = 0.033), and the rate of ATC was higher in the malignant tumor group. All patients diagnosed with lymphoma were included in the geriatric group (*p* = 0.001). In particular, patients diagnosed with ATC diagnosis had more frequent lymph node involvement (*p* = 0.016), extrathyroidal extension (*p* = 0.001), perineural invasion (*p* < 0.001), advanced pT stage and larger tumor size (*p* < 0.001). It is important to examine thyroid cancer types and associated histopathological parameters across different age groups. This is due to the fact that the incidence of thyroid cancer tends to increase with age, and advanced disease stages, often linked with unfavorable histopathological parameters, could potentially contribute to higher rates of thyroid cancer-specific deaths [13].

The prognostic importance of patient age at the time of diagnosis has long been known. More than 50% of all cancers occur in patients >65 years, making age a poor prognostic factor for cancer [12]. In some studies, male sex, increased tumor size, presence of a pT3–4 tumor, lymph node metastasis, and the incidence of distant metastasis have been demonstrated to be higher in geriatric patients who underwent thyroidectomy [23,24]. In another study, multifocality was identified as an important risk factor for recurrence [25]. Meanwhile, a different study found that increased tumor size, extrathyroidal extension, lymph node involvement and BRAF V600E mutations were major risk factors for poor prognosis [15]. Approximately 16% of patients in our study were geriatric. Considering the characteristics of histopathological parameters in the geriatric group, a significant correlation was found between increased tumor size (*p* < 0.001) and more advanced pT stage (*p* = 0.001), and 68% of patients were male (*p* = 0.014). When considering the histopathological parameters according to sex, the rate of malignant tumor diagnosis was higher in males, who also exhibited a more advanced pT stage (*p* < 0.001), larger tumor size (*p* < 0.001), and increased lymph node involvement (*p* = 0.039). This may be due to the late diagnosis of geriatric patients. There is a general belief that surgical treatment for thyroid diseases in geriatric patients is risky due to their advanced age and given comorbidities. For this reason, patients are frequently followed up with conservative treatment methods such as medication or radioactive iodine therapy. However, delayed surgical treatment may expose elderly patients to undesirable risks such as metastasis and an aggressive clinical course, especially in the context of malignant disease [21]. At this point, it may be important to undergo an ultrasound on these patients in order to observe changes in known thyroid lesions [26]. Clinicians evaluating elderly patients with thyroid nodules need to weigh the risks and benefits of clinical and surgical treatment strategies when deciding the next step of treatment [5]. Understanding the morbidity and mortality of thyroid surgery in this patient population is also essential [1]. Given the higher malignancy rates and adverse histopathological parameters in the male geriatric group, these findings suggest a potentially poorer prognosis, especially in males. Consequently, thyroidectomy should be considered primarily in these patients.

In a review of studies categorizing thyroid cancer patients by age, one such study revealed that the rate of thyroidectomy decreases with advancing geriatric age and also found larger tumor size, higher multifocality rate, advanced stage tumor, metastasis and extracapsular invasion rate in the mid-elderly patient group compared to the non-geriatric and the young-elderly group [27]. In another study, extracapsular extension was more common in the mid-elderly group, and no difference was found in terms of tumor size, vascular invasion, lymph node involvement and distant metastasis [15]. In our study, we noted a significantly higher distribution ratio of males in the young-elderly group (*p* = 0.004). When assessing the characteristics of histopathological parameters according to geriatric age groups, we found that the incidence of extrathyroidal extension (*p* < 0.001), lymph node involvement (*p* = 0.005), surgical margin involvement (*p* = 0.029) and pT3–4 stage tumor (*p* < 0.001) was notably prevalent in the mid-elderly geriatric age group. The safety of thyroidectomy in elderly patients is still a controversial topic in the literature. Although there is no consensus on this issue, surgery is less frequently recommended for elderly patients due to increased operative risks [21]. In our study, the decrease in the rate of thyroidectomy with increasing geriatric age seems to be reflective of this trend. Age-related, more aggressive histopathological features may be associated with delayed diagnosis in the elderly [28]. Multiple factors should be considered when making treatment decisions for thyroid diseases in geriatric patients. If malignancy is suspected, particularly in male patients in this age range, thyroidectomy might be prioritized over medical treatment, depending on the patient’s other conditions, comorbidities and overall vitality. In such scenarios, age should not be viewed as an absolute risk factor but rather as a risk factor related to the patient’s comorbidities and general clinical condition.

## 6. Conclusions

The safety of thyroidectomy in geriatric populations remains controversial. Surgical safety is of particular concern for older patients due to the increased prevalence of thyroid disease with advancing age. This study focused on the current knowledge of histopathological parameters related to thyroid surgery in older adults to help inform decision-making among elderly patients and their physicians. Although benign thyroid diseases are more common in the geriatric population, aggressive forms of malignant tumors can be seen more frequently. Thyroidectomy should be seriously considered in the treatment of these patients, especially in males, as the rate of malignant diagnosis and worse histopathological features rises with increasing age. As the incidence of unfavorable histopathological parameters heightens with geriatric age, surgical treatment should not be relegated to a secondary option solely due to the patient’s advanced age. Instead, surgical intervention should be primarily considered after a thorough evaluation of the patient’s comorbidities and well-being.

## 7. Limitations and the Shortcomings

A major limitation of the study is that only patients who actually had thyroid surgery were considered. Since the study focused on thyroidectomy specimens and their evaluation criteria, no attempt has been made to investigate how many older patients in the institution were found to have thyroid disorders. Another limitation of our study includes an insufficient sample size within the advanced-elderly geriatric age group, preventing a statistically meaningful evaluation of the examined histopathological parameters in this demographic. For this reason, our study cannot provide meaningful results for the advanced-elderly group. In our hospital, thyroidectomy seems to be less favored as a treatment option for advanced geriatric patients. That is why, in order to decide on the most appropriate treatment for the elderly population, larger case groups comprising a statistically adequate number of the elderly group are needed. Moreover, further studies, including a comprehensively broader sample size, comparing the differences between geriatric patients and adults, malignant histopathological features and a multivariate analysis are required.

## Figures and Tables

**Table 1:** Demographic and histopathological characteristics of the patients included in the study by sex.

	Male (%)	Female (%)	N (%)	*p*-Value
Mean age (years)	53.2 ± 13.7	48.6 ± 14.5	49.7 ± 14.4	**<0.001**
Age intervals				**0.004**
Under 25	8 (0.8)	44 (4.8)	52 (5.7)	
25–34 years	15 (1.6)	82 (9)	97 (10.6)	
35–44 years	35 (3.8)	145 (15.9)	180 (19.7)	
45–54 years	43 (4.7)	163 (17.9)	206 (22.6)	
55–64 years	68 (7.4)	163 (17.9)	231 (25.3)	
65–74 years	41 (4.5)	78 (8.5)	119 (13)	
75–84 years	5 (0.5)	18 (2)	23 (2.5)	
85 years and above	0	2 (0.2)	2 (0.2)	
Geriatric grouping				**0.014**
Under 65 (Non-geriatric)	169 (18.5)	597 (65.7)	766 (84.2)	
65 years and above (Geriatric)	46 (5)	98 (10.7)	144 (15.8)	
Surgery procedure				0.096
Subtotal thyroidectomy	51 (5.6)	129 (14.2)	180 (19.8)	
Total thyroidectomy	164 (18)	566 (62.2)	730 (80.2)	
Histopathological group				0.957
Benign tumors	146 (16)	471 (51.8)	617 (67.8)	
Low-risk tumors	5 (0.5)	14 (1.5)	19 (2.1)	
Malignant tumors	64 (7)	210 (23)	274 (30.1)	
Tumor size	2.04 ± 1.82	1.33 ± 1.06	1.5 ± 1.32	**<0.001**
Tumor localization				0.341
Right lobe	57 (13.7)	186 (44.7)	243 (58.4)	
Left lobe	45 (10.8)	120 (28.8)	165 (39.6)	
Isthmus	0	8 (1.9)	8 (1.9)	
Multifocality				0.637
Available	21 (7.1)	75 (25.6)	96 (32.7)	
Absent	48 (16.4)	149 (50.8)	197 (67.3)	
Lymphovascular invasion				0.811
Available	22 (7.5)	59 (20.1)	81 (27.6)	
Absent	47 (16)	165 (56.3)	212 (72.4)	
Perineural invasion				0.067
Available	9 (3)	14 (4.7)	23 (7.8)	
Absent	60 (20.4)	210 (71.6)	270 (92.1)	
Surgical margin status				0.238
Available	8 (2.7)	16 (5.4)	24 (8.2)	
Absent	61 (20.8)	208 (71)	269 (91.7)	
Extrathyroidal extension				0.359
Available	5 (1.7)	10 (3,4)	15 (5.3)	
Absent	64 (21.8)	214 (73)	278 (94.8)	
Lymph node involvement				0.390
Available	16 (5.4)	29 (9.9)	45 (15.3)	
Absent	53 (18.1)	195 (66.5)	248 (84.7)	
pT Stage				**0.001**
pT 1	40 (14.7)	170 (62.5)	210 (77.2)	
pT 2	10 (3.6)	27 (9.9)	37 (12.5)	
pT 3	12 (4.4)	9 (3.3)	21 (7.7)	
pT 4	2 (0.7)	2 (0.7)	4 (1.5)	
Total	215 (23.6)	695 (76.4)	910 (100)	

**Table 2:** Demographic and histopathological characteristics of the patients according to geriatric grouping.

	Geriatric Group (%)	Non-Geriatric Group (%)	N (%)	*p*-Value
Sex				**0.010**
Female	98 (10.7)	597 (65.7)	695 (76.5)	
Male	46 (5)	169 (18.5)	215 (23.5)	
Surgery procedure				0.211
Subtotal thyroidectomy	23 (2.5)	157 (17.2)	180 (19.8)	
Total thyroidectomy	121 (13.3)	609 (66.8)	730 (80.2)	
Histopathological group				0.422
Benign tumors	104 (11.4)	513 (56.3)	617 (67.8)	
Low-risk tumors	2 (0.2)	17 (1.8)	19 (2.1)	
Malignant tumors	38 (4.1)	237 (26.1)	274 (30.1)	
Tumor size	2.25 ± 2.15	1.38 ± 1.10	1.5 ± 1.32	**<0.001**
Tumor localization				0.557
Right lobe	30 (7.2)	213 (51.2)	243 (58.4)	
Left lobe	20 (4.8)	145 (34.8)	165 (39.6)	
Isthmus	2 (0.4)	6 (1.4)	8 (1.9)	
Multifocality				0.260
Available	10 (3,4)	86 (29.3)	96 (32.7)	
Absent	30 (10.2)	167 (57)	197 (67.3)	
Lymphovascular invasion				0.162
Available	10 (3,4)	71(24.2)	81 (27.6)	
Absent	30 (10.2)	182 (62.1)	212 (72.4)	
Perineural invasion				0.239
Available	5 (1.7)	18 (6.1)	23 (7.8)	
Absent	35 (11.9)	235 (80.1)	270 (92.1)	
Surgical margin status				0.285
Available	5 (1.7)	19 (6.5)	24 (8.2)	
Absent	35 (11.9)	234 (80.7)	269 (91.7)	
Extrathyroidal extension				0.132
Available	4 (1.3)	11 (3.7)	15 (5.2)	
Absent	36 (12.3)	242 (82.6)	278 (94.8)	
Lymph node involvement				0.291
Available	5 (1.7)	40 (13.6)	45 (15.3)	
Absent	35 (11.9)	213 (72.7)	248 (84.7)	
pT Stage				**0.001**
pT 1	24 (8.8)	186 (68.3)	210 (77.1)	
pT 2	4 (1.4)	33 (12.1)	37 (13.5)	
pT 3	5 (1.8)	16 (5.8)	21 (7.7)	
pT 4	3 (1.1)	1 (0.3)	4 (1.4)	
Total	144 (15.8)	766 (84.2)	910 (100)	

**Table 3:** Demographic and histopathological characteristics of the patients according to age groups.

	<25	25–34	35–44	45–54	55–64	65–74	75–84	≥85	*p*-Value
Sex									**0.004**
Female	44	82	145	163	163	78	18	2	
Male	8	15	35	43	68	41	5	0	
Surgery procedure									0.416
Subtotal thyroidectomy	19	20	34	32	44	21	2	0	
Total thyroidectomy	33	77	140	172	187	98	21	2	
Histopathological group									0.508
Benign tumors	34	57	128	133	160	89	14	1	
Low-risk tumors	0	2	4	4	7	2	0	0	
Malignant tumors	18	38	48	69	64	28	9	1	
Tumor size	1.62 ± 1.26	1.28 ± 0.74	1.36 ± 1.04	1.44 ± 1.32	1.34 ± 1.05	1.66 ± 1.76	3.06 ± 2.73	7	0.402
Multifocality									0.639
Available	8	14	13	28	23	7	3	0	
Absent	10	26	38	45	48	23	6	1	
Bilaterality									0.446
Available	7	9	11	20	14	4	1	0	
Absent	11	31	40	53	57	26	8	1	
Lymphovascular invasion									0.209
Available	9	12	11	18	21	4	3	1	
Absent	9	28	40	55	50	26	6	0	
Perineural invasion									0.656
Available	0	3	3	7	5	3	2	0	
Absent	18	37	48	66	66	27	7	1	
Surgical margin status									**0.029**
Available	1	3	3	8	4	2	2	1	
Absent	17	37	48	65	67	28	7	0	
Extrathyroidal extension									**<0.001**
Available	1	4	2	2	2	1	2	1	
Absent	17	36	49	71	69	29	7	0	
Lymph node involvement									**0.005**
Available	8	9	8	8	7	2	3	0	
Absent	10	31	43	65	64	28	6	1	
pT staging									**<0.001**
pT 1	13	32	39	50	52	19	5	0	
pT 2	2	4	7	13	7	3	1	0	
pT 3	3	2	1	6	4	5	0	0	
pT 4	0	0	0	0	1	0	2	1	
Total	52	97	180	206	231	119	23	2	

## Data Availability

Data are available upon reasonable request from the corresponding authors.
